# A Phylogenetic Analysis Based on Whole Genome Re-Sequencing of 41 *Dendrobium* Species

**DOI:** 10.3390/cimb47040276

**Published:** 2025-04-15

**Authors:** Feng-Ping Zhang, Xue-Wei Fu, Han-Run Li, Shi-Bao Zhang

**Affiliations:** 1 College of Traditional Chinese Medicine, Yunnan Key Laboratory of Dai and Yi Medicines, Yunnan University of Chinese Medicine, Kunming 650500, China; 2 Key Laboratory of Economic Plants and Biotechnology, Yunnan Key Laboratory for Wild Plant Resources, Kunming Institute of Botany, Chinese Academy of Sciences, Kunming 650201, China; 3 Kunming Botanical Garden, Kunming Institute of Botany, Chinese Academy of Sciences, Kunming 650201, China

**Keywords:** whole genome re-sequencing, *Dendrobium*, authentication, phylogenetic relationships, divergence time

## Abstract

The genus *Dendrobium* (Orchidaceae) is highly renowned for its great medicinal and ornamental values. However, due to morphological similarities among closely related taxa within this genus, certain species are frequently subject to misidentification and adulteration in the market. Traditional morphological taxonomy and limited DNA markers prove challenging in effectively differentiating among them. Here, we generated an extensive single nucleotide polymorphism (SNP) dataset through whole genome re-sequencing (WGRS) of 41 *Dendrobium* species to evaluate its effectiveness in species identification. The phylogenetic relationships of 41 *Dendrobium* species were explored based on the SNP dataset, and then divergence times at each node were estimated. We found that the whole genome re-sequencing method achieved a 100% identification rate for all 41 species examined, indicating that whole genome re-sequencing could be employed to accurately authenticate *Dendrobium* species. Furthermore, phylogenetic analysis revealed that the sect. *Dendrobium* was polyphyletic. In addition, the divergence time analysis suggested that *Dendrobium* originated since the Oligocene. These findings provide valuable genetic data resources for further systematic studies of the rare and endangered *Dendrobium* species.

## 1. Introduction

Accurate species identification is the basis for biodiversity conservation and sustainable use. However, effectively identifying species is not an easy task. Traditional methods for identifying closely related species based on morphological characteristics can be difficult due to similar morphology, especially for some species that do not have diagnostic organs or characters, such as flowers [1,2,3,4,5]. Whole genome re-sequencing is an efficient molecular method that uses single nucleotide polymorphism (SNP) dataset for accurate species identification, with the advantage of more sufficient data and higher resolution, and has become a powerful approach for species identification, cryptic species discovery, and conservation biology of endangered species, etc. [6,7]. *Dendrobium* is an orchid genus that has great medicinal and ornamental value. Its taxonomy is considered one of the most difficult problems in the family Orchidaceae, due to the similar morphological characteristics of species, especially during the vegetative phase (non-flowering stage) [3,4,5]. As a result, misidentifications between *Dendrobium* species on the market are common [8,9,10]. For example, *D. officinale* has often been confused with other *Dendrobium* species because they are so similar in morphology [9,10,11].

In recent years, molecular methods have been increasingly used for the identification of *Dendrobium* species. Clements (2006) utilized nrDNA *ITS* sequence data to evaluate the molecular phylogenetic systematics of *Dendrobium* species, in which sect. Calcarifera, *Dendrobium*, *Oxyglossum*, *Pedilonum*, and *Rhopalanthe* were all shown to be either paraphyletic or polyphyletic [12]. A combination of five genomic hotspots, namely *trnT*-*trnL*, *rpl32*-*trnL*, *clpP*-*psbB*, *trnL* intron, and *rps16*-*trnQ*, have been demonstrated to be useful in phylogenetic and identification studies within this genus [13]. Complete plastome sequences have been successfully applied to accurately authenticate *D. officinale* and distinguish it from its closely related species [14]. Although several well-supported clades have been identified, there remain a number of species that do not nest inside any of these clades. For instance, closely related species such as *D. lindleyi* and *D. jenkinsii*, along with *D. senile*, *D. trigonopus*, and *D. capillipes* were considered unplaced based on sequences of the Internal Transcribed Spacer 1 (partial), 5.8S ribosomal RNA gene (complete), and Internal Transcribed Spacer 2 (partial) [15]. Moreover, *D. capillipes*, and *D. trigonopus* are still unplaced based on the sequences of *rbcL*, *matK*, *trnH-psbA* spacer, and *trnL* intron [16].

Although there is a certain degree of consensus regarding the molecular systematics of *Dendrobium*, previous studies have largely concentrated on a single section and mainly employed either a single DNA marker or multiple molecular markers [16]. This has resulted in a low level of support for some conclusions and findings. Consequently, there is a need to gain a more comprehensive understanding of the phylogenetic relationships within this genus and the taxonomic delimitation using more extensive molecular evidence. The whole genome re-sequencing method presents an opportunity to distinguish closely related species. However, few studies have utilized whole genome re-sequencing data to identify *Dendrobium* species. In this study, we re-sequenced the genomes of 41 *Dendrobium* species. Our specific objectives were as follows: (1) to detect whether the single nucleotide polymorphism (SNP) dataset by whole genome re-sequencing is suitable for discriminating *Dendrobium* species; and (2) to infer and test the phylogenetic relationships and divergence times among species using whole genome re-sequencing.

## 2. Materials and Methods

### 2.1. Plant Materials, Sampling and Whole Genome Resequencing

**Figure 1:**
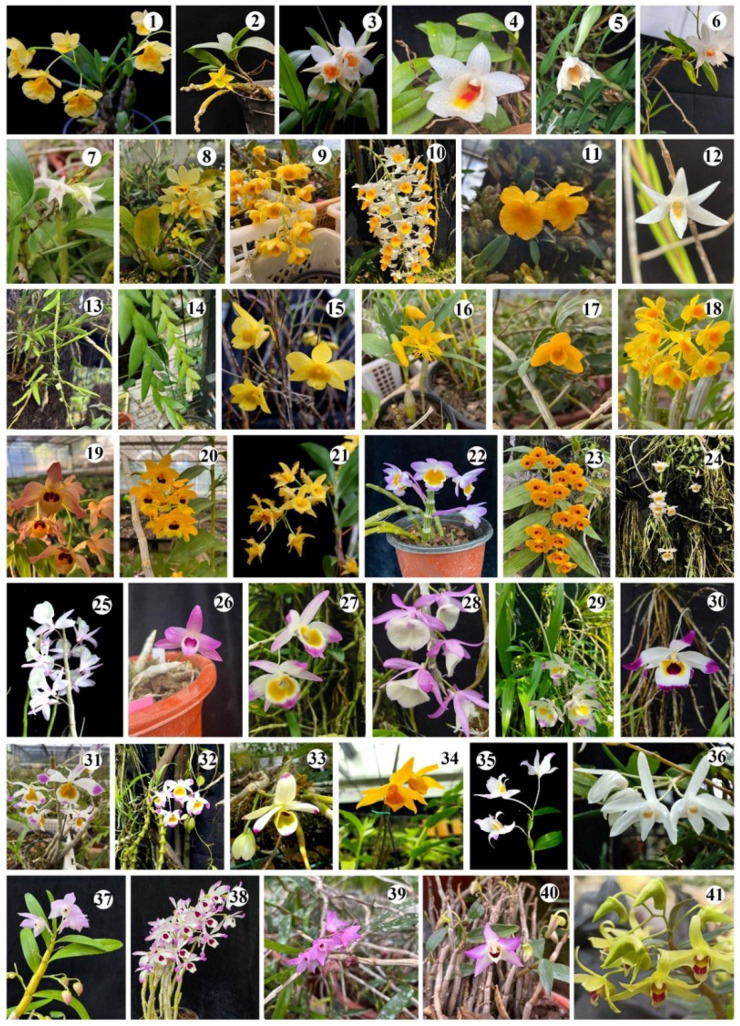
The 41 *Dendrobium* species sampled in this study. (1) *D. capillipes*, (2) *D. trigonopus*, (3) *D. cariniferum*, (4) *D. christyanum*, (5) *D. longicornu*, (6) *D. wattii*, (7) *D. stuposum*, (8) *D. sulcatum*, (9) *D. densiflorum*, (10) *D. thyrsiflorum*, (11) *D. jenkinsii*, (12) *D. exile*, (13) *D. spatella*, (14) *D. terminale*, (15) *D. hancockii*, (16) *D. brymerianum*, (17) *D. lohohense*, (18) *D. chrysotoxum*, (19) *D. moschatum*, (20) *D. fimbriatum*, (21) *D. harveyanum*, (22) *D. crepidatum*, (23) *D. chrysanthum*, (24) *D. loddigesii*, (25) *D. aphyllum*, (26) *D. parishii*, (27) *D. gratiosissimum*, (28) *D. polyanthum*, (29) *D. devonianum*, (30) *D. falconeri*, (31) *D. crystallinum*, (32) *D. pendulum*, (33) *D. wardianum*, (34) *D. henryi*, (35) *D. findlayanum*, (36) *D. moniliforme*, (37) *D. hercoglossum*, (38) *D. nobile*, (39) *D. aduncum*, (40) *D. flexicaule*, and (41) *D. officinale*.

Plants of 41 *Dendrobium* species were grown in a greenhouse at the Kunming Institute of Botany, Chinese Academy of Sciences (Kunming, China; 25°10ʹ N, 102°41ʹ E). (Figure 1, Table S1). Voucher specimens were deposited in the Herbarium of Kunming Institute of Botany, Chinese Academy of Sciences (the number of vouchers are shown in Table S1). The total genomic DNA of each sample was extracted from fresh and healthy leaves using the modified CTAB method [17]. The quality of the extracted DNA was examined using a NanoDrop 2000 spectrophotometer (Thermo Fisher Scientific, Waltham, MA, USA) and its quantity was determined by electrophoresis on a 0.8% agarose gel. The diluted working solution of DNA was stored at 4 °C, while the storage solution was stored at −20 °C. Illumina sequencing libraries were generated using Illumina’s TruSeq DNA PCR-free prep kits (Illumina, San Diego, CA, USA) following the protocol of the manufacturer. The total DNA was fragmented by the Covaris system and then processed and sequenced on Illumina NovaSeq 6000 (Illumina, San Diego, CA, USA) platforms. The library was quantified using a Bioanalyzer 2100 system (Illumina, San Diego, CA, USA). The raw reads were filtered to obtain high quality reads by Fastp (version: 0.20.0) [18]. Paired reads containing over 10% N and/or having a base quality ≤50 were removed. The remaining reads were regarded as clean data.

### 2.2. Whole Genome Sequence Alignment, SNP Detection, and Annotation

Clean paired-end reads from all samples were mapped to the *D. nobile* reference genome (accession number: GCA 022539455) using BWA-mem 0.7.17-r11188 with default parameters. The alignment files were then converted into BAM format and sorted by SAMtools (v.0.1.18) with default settings [19]. Duplicate reads were marked and removed using MarkDuplicates in Picard 1.107. Subsequently, only single nucleotide polymorphism (SNP) variants were selected for further analyses. SNPs were detected using GATK 3.8 [20], and the SNP Loci were annotated using ANNOVAR (version: 2017Jul17) [21].

### 2.3. Phylogenetic Analyses and Divergence Time Estimation of Dendrobium

The phylogenetic tree was inferred from 147,907,696 single nucleotide ploymorphisms (SNPs) using the Bayesian inference (BI) method. *Bletilla striata* was used as the outgroup to root the phylogenetic tree. MrBayes 3.2.7a software [22] was employed for the BI analysis. This analysis involved two independent Markov Chain Monte Carlo (MCMC) runs, each consisting of two million generations.

The divergence time was estimated by implementing a Bayesian uncorrelated relaxed-clock model in MCMCTree (version 4.10.6) [23]. Three priors were used: (1) the stem age of *Dendrobium* was set at 31 Ma (26.92–34.03 Ma) (the root of the tree) [24]; (2) 23.2 Ma (15.9–29.3 Ma) as the age for *D. cariniferum* with *D. trigonopus* [25]; and (3) 8.3 Ma (5.9–11.4 Ma) as the age for *D. chrysanthum* with *D. crepidatum* [24]. All priors were set under the normal distribution. The Markov Chain Monte Carlo (MCMC) searches were run for 50,000,000 generations with sampling every 10,000 generations. Convergence was monitored using Tracer 1.6 [26].

## 3. Results

### 3.1. Species Discrimination of Dendrobium Based on Phylogenetic Tree

**Figure 2:**
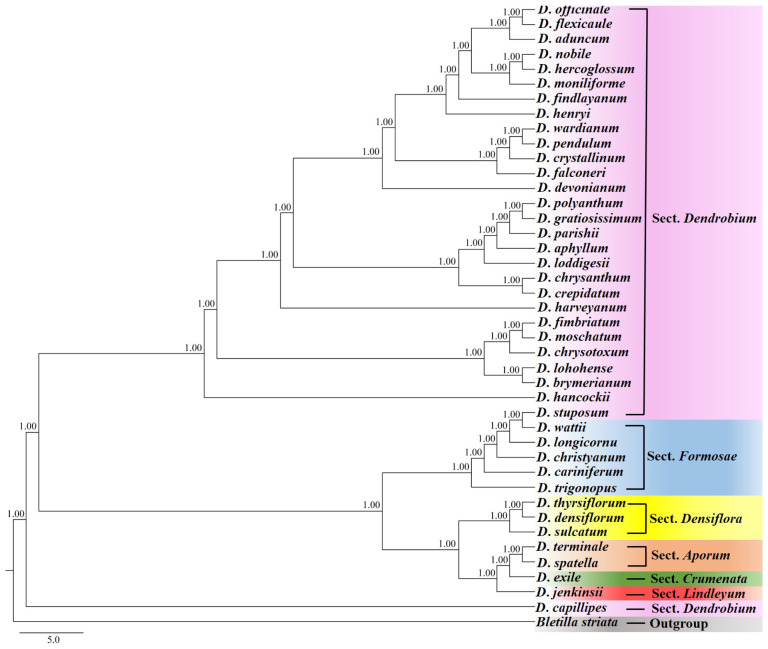
Bayesian inference (BI) phylogeny of 41 *Dendrobium* species analyses based on single nucleotide polymorphism (SNP) by whole genome re-sequencing. Numbers at nodes indicate the BI posterior probabilities (PP).

In this study, the single nucleotide polymorphism (SNP) dataset obtained from whole genome re-sequencing was used for species identification analysis; the results demonstrated that the phylogenetic relationships of all 41 *Dendrobium* species were resolved through Bayesian inference (BI) phylogeny with robust support values (PP = 1.00) (Figure 2). All relationships among the major clades were strongly supported, indicating that the SNP dataset achieved a high species identification rate. Specifically, all 41 species were successfully identified, accounting for 100%. The sect. *Dendrobium* consisted of 29 species. The sect. *Aporum* contained two species; one species was from sect. *Crumenata*, and another species was from sect. *Lindleyum*. Three species belonged to sect. *Densiflora*, five species were from sect. *Formosae* (Figure 2). *D. capillipes* formed a branch as a species of sect. *Dendrobium*; *D. trigonopus* is nested within sect. *Formosae* with a high support rate (PP = 1.00). Meanwhile, *D. jenkinsii* was classified as belonging to sect. *Lindleyum*.

### 3.2. Divergence Time Estimation

**Figure 3:**
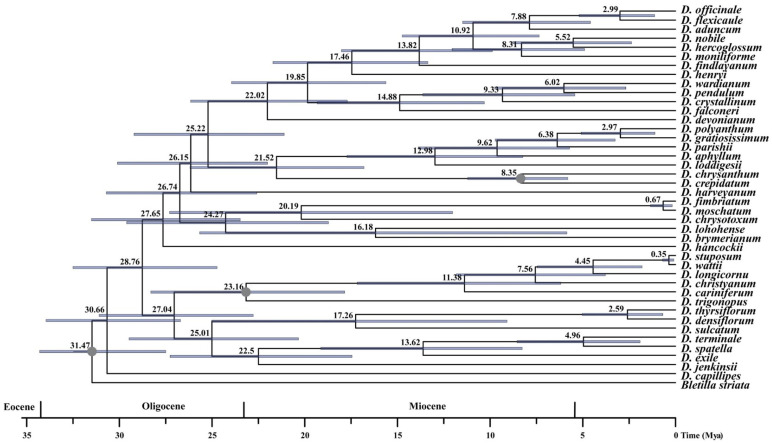
The divergence time estimation from the Bayesian inference (BI) phylogenetic tree of 41 *Dendrobium* species analyses based on single nucleotide polymorphism (SNP) by whole genome re-sequencing. The blue bars correspond to the 95% highest posterior density (HPD).

The divergence time analysis of *Dendrobium* using the SNP data indicated that the stem and crown ages of *Dendrobium* were 31.47 Ma (95% highest posterior density (HPD): 27.49–34.30 Ma) and 30.66 Ma (95% HPD) in the Oligocene, respectively (Figure 3). The majority of species from sect. *Dendrobium* had a stem age of 28.76 Ma and a crown age of 27.65 Ma, respectively, both occurring in the middle Oligocene. Sect. *Densiflora* diverged from sect. *Aporum*, sect. *Crumenata*, and sect. *Lindleyum* at 25.01 Ma, during the late Oligocene. The sect. *Linleyum*, sect. *Crumenata*, and sect. *Aporum* split apart at 22.5 Ma during the early Miocene. The split between sect. *Aporum* and sect. *Crumenata* occurred at 13.62 Ma, during the middle Miocene.

## 4. Discussion

The genus *Dendrobium* is an important genus, comprising well-known herbaceous medicinal plants that are utilized as diverse herbal medicines for treating various diseases [27]. However, *Dendrobium* is a taxonomically complex group due to its stem convergent morphology and the close phylogenetic relationships [4,16,28,29,30,31]. Traditionally, *Dendrobium* has been classified mainly on the basis of morphological features. Nevertheless, natural hybridization within the genus leads to insufficient diagnostic traits for accurate authentication [4,31]. As a result, genetic techniques have been introduced to distinguish these important herbal medicines.

Previous molecular identification studies based on one or a few DNA regions were also proven to be ineffective in authenticating certain *Dendrobium* species [32,33,34]. Neither single markers nor combinations of multi-markers were capable of identifying all the tested *Dendrobium* species [35]. For instance, the phylogenetic relationships of five species, i.e., *Dendrobium capillipes*, *D. trigonopus*, *D. senile*, *D. lindleyi*, and *D. jenkinsii*, remained poorly resolved with weak support when using plastid *rbcL*, *matK*, *trnH-psbA* spacer and *trnL* intron and nuclear *ITS* sequences [16]. Complete plastome sequences were used to distinguish *Dendrobium officinale* from its closely related species, demonstrating the significant advantages of these sequences in differentiating *Dendrobium* species [14]. Recently, with the utilization of complete plastome sequences and a relatively broad sampling scale, 23 *Dendrobium* species were identified [35].

However, the efficacy of other methods in authenticating other taxonomically complex *Dendrobium* taxa remains to be assessed. Single nucleotide polymorphism (SNP) markers obtained from whole genome re-sequencing have characteristics such as abundance, wide distribution, high genetic stability, low analysis cost, and strong tolerance to DNA degradation [36,37]. In recent years, whole genome re-sequencing has been extensively applied in population genetic analysis, species identification, exploration of genes associated with important functional traits, and conservation biology of endangered plants [10,38,39,40,41,42,43]. In this study, all of the 41 *Dendrobium* species were effectively differentiated using the single nucleotide polymorphism (SNP) dataset obtained through whole genome re-sequencing. The phylogenetic relationships of all the tested species were clearly resolved with high support rates. Our results confirmed the effectiveness of whole genome re-sequencing in the identification of *Dendrobium* species. Thus, the application of whole genome re-sequencing is a promising way for authenticating species, particularly those that are taxonomically and phylogenetically complex.

*Dendrobium officinale* has often been regarded as a member of the *D. moniliforme* complex [44]. However, our findings demonstrated that it clustered within the branch containing *D. flexicaule* and *D. aduncum* with strong support (PP = 1.00), which is in line with earlier studies [16,35]. The phylogenetic relationship within the Asian *Dendrobium* clade has been much more confusing [15]. Despite the presence of several well-supported clades, there were numerous species that did not nest within any of these clades. For example, *D. capillipes*, *D. trigonopus*, and *D. jenkinsii*, which were considered unplaced [16]. Based on five DNA markers and a broad sampling of *Dendrobium*, the results suggested that *D. jenkinsii* might represent a distinct section, while the former two species, *D. capillipes* and *D. trigonopus*, remained unclassified [16]. Morphologically, *D. capillipes* is a typical species of sect. *Dendrobium* [16,45], and *D. trigonopus* does not belong to sect. *Formosae* [16]. Our analysis indicated that *D. capillipes* formed a branch as a species of sect. *Dendrobium*, corroborating previous results [15,45]. *D. trigonopus* was nested within sect. *Formosae* with a high support rate (PP = 1.00). A new section (sect. *Lindleyum*) had been proposed for *D. jenkinsii* [16], and this is supported by the current findings.

The results of the divergence time analysis indicated that *Dendrobium* originated in the Oligocene. The discovery of *Dendrobium* leaf fossils in New Zealand suggested the expansion of *Dendrobium* into Zealandia during the middle Cenozoic (Early Miocene, 23–20 Ma) [46]; this expansion time is younger than our study’s estimate, as the crown age of *Dendrobium* in our analysis diverged at 30.66 Ma. However, the crown age estimate of this genus by Xiang et al., (2016) [24] was 28.17 Ma, which is similar to our result. The rapid diversification of *Dendrobium* species occurred from the late Oligocene to middle Miocene; this finding is consistent with the result of a previous study [24], and also coincides with the period of global warming [24,47,48,49,50].

## 5. Conclusions

In summary, this study represents the first attempt to identify the taxonomically challenging *Dendrobium* species group through whole genome re-sequencing. The results demonstrated that the SNP dataset obtained from the whole genome re-sequencing exhibited a remarkable discriminatory power of 100% (PP = 1.00) for 41 *Dendrobium* species examined. We suggest that whole genome re-sequencing could be effectively employed to accurately distinguish *Dendrobium* species. This research will significantly facilitate investigations into the evolutionary and phylogenetic relationships of *Dendrobium*. Moreover, it broadens our understanding of plant diversity, contributing to the broader field of botanical research.

## Data Availability

The data and materials in this study are available from the corresponding author upon reasonable request.

## Supplementary Materials

The following supporting information can be downloaded at: https://www.mdpi.com/article/10.3390/cimb47040276/s1.
