# Supplementary material for: A Phylogenetic Analysis Based on Whole Genome Re-Sequencing of 41 Dendrobium Species
Source: Curr Issues Mol Biol. 2025 Apr 15;47(4):276. doi: 10.3390/cimb47040276 (PMC12025457; doi:10.3390/cimb47040276)
Supplement: Supplementary file 1 [file cimb-47-00276-s001.zip › cimb-3494212-supplementary.pdf]

Supporting information

Table S1 The sampled 41 *Dendrobium* species

Species	Section	Voucher number	Coverage
D. capillipes	*Dendrobium*	1630734	32.77%
D. trigonopus	*Formosae*	1630723	37.37%
D. cariniferum	*Formosae*	1630736	34.26%
D. christyanum	*Formosae*	1630746	34.83%
D. longicornu	*Formosae*	1630725	34.50%
D. wattii	*Formosae*	1630758	34.11%
D. stuposum	*Dendrobium*	1630739	35.04%
D. sulcatum	*Densiflora*	1630752	35.25%
D. densiflorum	*Densiflora*	1630724	35.48%
D. thyrsiflorum	*Densiflora*	1630756	36.43%
D. jenkinsii	*Lindleyum*	1630744	33.46%
D. exile	*Crumenata*	1630731	25.45%
D. spatella	*Aporum*	1630743	26.72%
D. terminale	*Aporum*	1630742	26.43%
D. hancockii	*Dendrobium*	1630754	37.31%
D. brymerianum	*Dendrobium*	1630730	39.75%
D. lohohense	*Dendrobium*	1630750	39.92%
D. chrysotoxum	*Dendrobium*	1630740	41.50%
D. moschatum	*Dendrobium*	1630755	40.85%
D. fimbriatum	*Dendrobium*	1630759	39.62%
D. harveyanum	*Dendrobium*	1630733	40.97%
D. crepidatum	*Dendrobium*	1630722	39.40%
D. chrysanthum	*Dendrobium*	1630749	42.85%
D. loddigesii	*Dendrobium*	1630753	35.77%
D. aphyllum	*Dendrobium*	1630728	37.73%
D. parishii	*Dendrobium*	1630718	37.19%
D. gratiosissimum	*Dendrobium*	1630721	35.09%
D. polyanthum	*Dendrobium*	1630720	36.95%
D. devonianum	*Dendrobium*	1630751	51.81%
D. falconeri	*Dendrobium*	1630726	52.93%
D. crystallinum	*Dendrobium*	1630719	54.42%
D. pendulum	*Dendrobium*	1630738	58.79%
D. wardianum	*Dendrobium*	1630727	57.09%
D. henryi	*Dendrobium*	1630760	62.29%
D. findlayanum	*Dendrobium*	1630747	72.76%
D. moniliforme	*Dendrobium*	1630737	71.59%
D. hercoglossum	*Dendrobium*	1630761	74.62%
D. nobile	*Dendrobium*	1630735	89.77%
D. aduncum	*Dendrobium*	1630762	71.23%
D. flexicaule	*Dendrobium*	1630729	70.75%
D. officinale	*Dendrobium*	1630745	71.33%
